# Obstructive Constipation in Two Patients With Severe Osteogenesis Imperfecta and Acetabular Protrusio

**DOI:** 10.5435/JAAOSGlobal-D-21-00194

**Published:** 2022-01-04

**Authors:** Erin Stockwell, Maegen Wallace

**Affiliations:** From the University of Nebraska Medical Center, Omaha, NE (Dr. Stockwell and Dr. Wallace) and the University of Nebraska Medical Center, Children's Hospital and Medical Center Omaha (Dr. Wallace).; Correspondence to Dr. Stockwell: elstockwell3@gmail.com

## Abstract

Osteogenesis imperfecta (OI) is a rare genetic condition resulting in decreased bone density and bony deformity and a wide variety of extraskeletal manifestations. Acetabular protrusio and constipation are both commonly associated with OI. We present two cases of severe pelvic deformity resulting in mechanical colonic outlet obstruction, which were successfully treated with a colostomy. Colostomy as the definitive treatment of severe constipation in OI has not previously been reported in the literature.

Osteogenesis imperfecta (OI) is a rare genetic condition affecting approximately 1/15,000 births. Several genetic mutations involving COL1A1 and COL1A2 genes have been identified as causes of OI.^1^ Although all patients with OI suffer from decreased bone density and increased susceptibility to fractures, there is a wide spectrum of clinical manifestations and disease severity. The severity of skeletal deformation increases with the overall severity of disease; notable bowing of the long bones and acetabular protrusio are often seen and progress with age. Extraskeletal manifestations can also vary and include dental, ophthalmic, and gastrointestinal (GI) dysfunction.^1,2^ We present two cases of OI with severe pelvic deformity causing mechanical colonic obstruction and refractory constipation requiring a colostomy. Two case reports of mechanical colonic obstruction secondary to acetabular protrusio in OI have been previously published. One of those patients was treated with pelvic osteotomies, whereas the other managed their constipation with serial home enemas.^3,4^ No other cases of mechanical obstruction secondary to pelvic deformation or the utilization of a colostomy for the definitive treatment of refractory constipation in OI have been reported.

## Case 1

**Figure 1:**
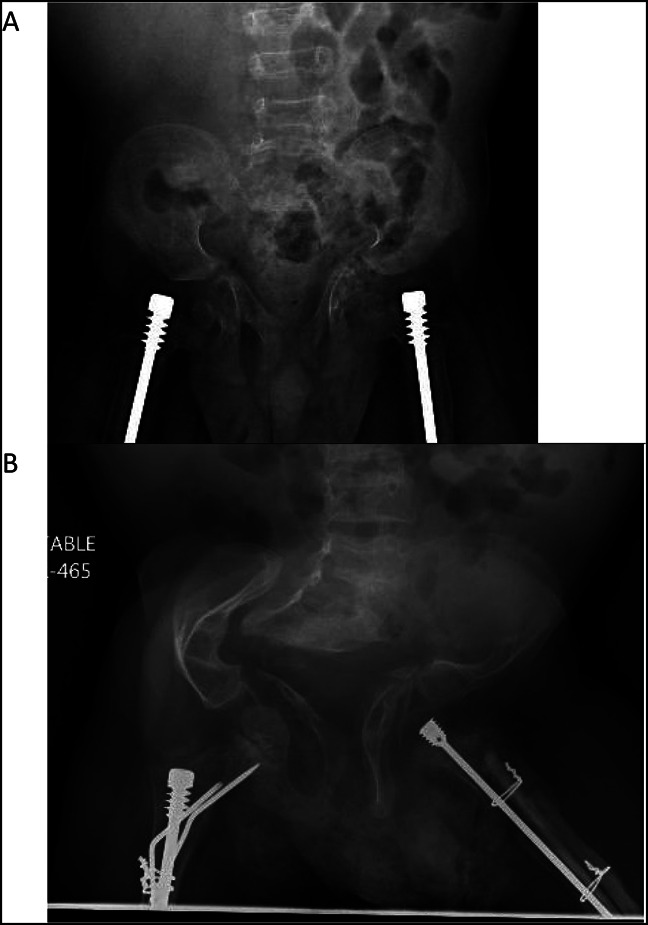
Radiograph of **A,** AP pelvis at 2 years and (**B**) AP pelvis at 8 years demonstrating marked progression of the patient's pelvic deformation and acetabular protrusio.

After sustaining multiple fractures in his first year of life, a 10-month-old male patient underwent genetic testing. A glycine to serine substitution was identified in the COL1A1 gene consistent with severe type 3 OI. He underwent his first orthopaedic surgery at 11 months of age at which time his lower extremity bowing was corrected, and Fassier-Duval intramedullary nails were placed in his bilateral femurs. He initiated pamidronate treatment shortly after this procedure. In addition to multiple long bone deformities, he developed progressively worsening pelvic deformation and bilateral acetabular protrusio, as shown in Figure 1.

The patient began experiencing constipation at 19 months of age. His symptoms were initially managed with oral stool softeners, motility agents, and rectal suppositories. His symptoms gradually became increasingly refractory and challenging to treat. Unfortunately, shortly before his eighth birthday, he was diagnosed with a severe *Clostridium difficile* infection which required hospitalization in the intensive care unit. Since this initial infection, he has had recurrent *C difficile* infections, often triggered from standard perioperative dosing of antibiotics. Given his severe and refractory constipation and recurrent *C difficile* infections, the patient ultimately underwent colostomy at age 8 years 6 months. Since the placement of his colostomy nearly 2 years ago, he has had minimal abdominal pain, marked improvement in his constipation, and no *C difficile* infections.

## Case 2

**Figure 2:**
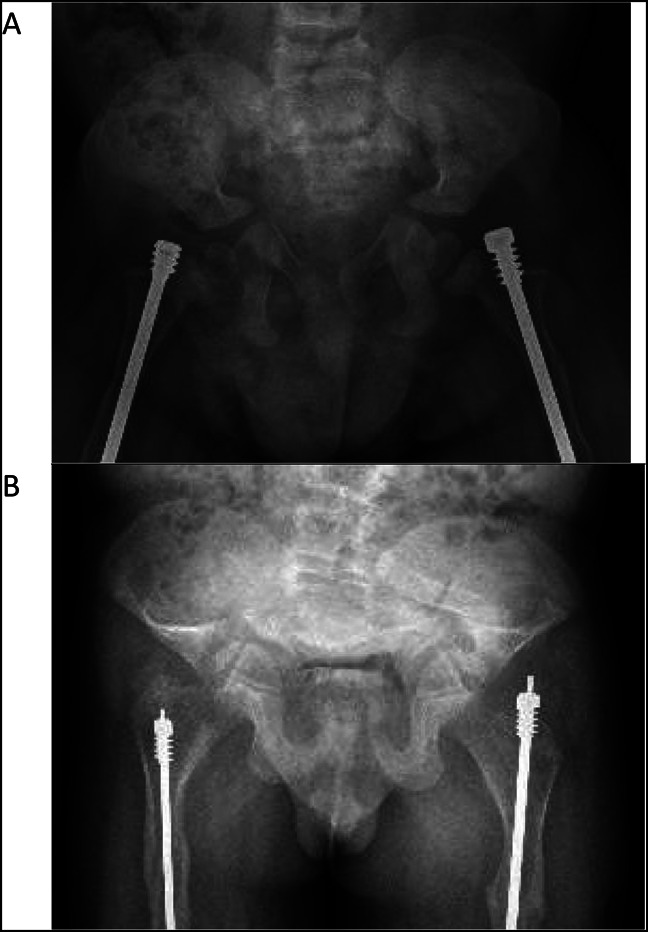
Radiograph of **A,** AP pelvis at 2 years and (**B**) AP pelvis at 10 years demonstrating marked progression of the patient's pelvic deformation and acetabular protrusio.

A 11-year-old boy was diagnosed with OI at 25 weeks of gestation. Genetic testing identified a homozygous mutation in the LEPRE1 gene at cDNA position c1345 with glycine to cysteine transition consistent with severe type 8 OI. He initiated pamidronate treatment shortly after birth but still experienced multiple fractures and upper respiratory infections during his first year of life. His first orthopaedic surgery occurred at 20 months of age at which time he underwent deformity correction and Fassier-Duval intramedullary nail placement in his left humerus. In addition to long bone deformities, he developed progressively worsening pelvic deformation and bilateral acetabular protrusion, as shown in Figure 2.

**Figure 3:**
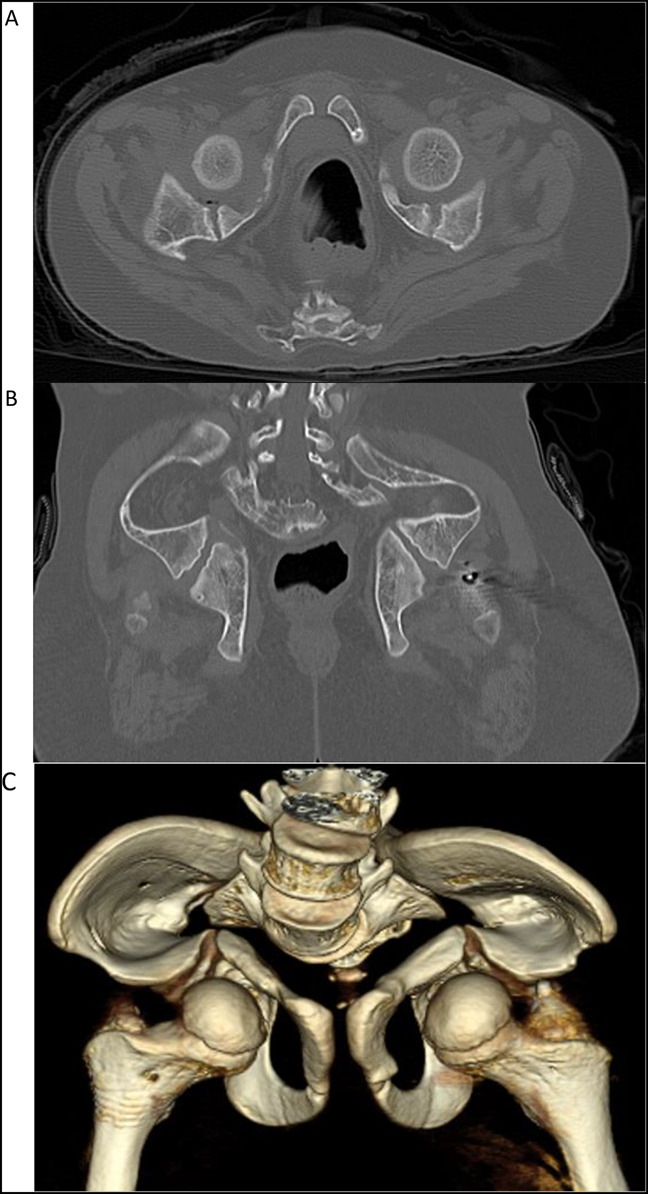
Radiograph showing **A,** axial and (**B**) coronal cuts along with (**C**) 3D reconstruction of pelvis CT obtained at 10 years.

The patient began experiencing severe constipation at age 10 years which was refractory to nearly all interventions. He underwent CT imaging (Figure 3) which demonstrated a mechanical colonic outlet obstruction secondary to his pelvic deformity. He ultimately underwent colostomy just before his 11th birthday. Since the placement of his colostomy nearly 1 year ago, he has had notable improvement in his constipation and associated abdominal pain.

## Discussion

Pelvic deformities and constipation are both commonly encountered in patients with severe OI. Acetabular protrusio has been reported in 33% to 55% of the patients with OI and in an estimated 70% of the patients specifically with type 3 OI.^5,6^ Although intuition suggests a correlation between acetabular protrusio and constipation, the limited data available have been inconsistent; Violas et al found that only 8% of patients with acetabular protrusio reported symptoms of constipation, whereas Lee et al demonstrated an increased risk of chronic constipation and recurrent abdominal pain in the presence of acetabular protrusio.^3,5^ No published studies exist that seek to assess the relationship between pelvic deformity and severity of constipation.

Constipation is one of the most commonly encountered extraskeletal symptoms in OI and has been reported in 26% to 58% of patients.^3,7^ The exact cause of constipation in patients with OI remains unclear. Intravenous diphosphonate therapy is often initiated in a young patient with OI to help improve bony quality.^1,2^ Although oral bisphosphonates have been reported to cause esophagus and upper GI symptoms, to the best of our knowledge, there are no reported cases of GI adverse effects with intravenous diphosphonate use.

In a single review of 43 patients with type 3 OI, 5 of the 11 patients with chronic constipation required fecal disimpaction.^7^ There is one published case report of a patient with OI who underwent colostomy for the treatment of megacolon and an additional case report of a patient with OI who underwent bilateral pelvic osteotomies for the treatment of colonic outlet obstruction secondary to their acetabular protrusio. In a brittle OI bone, pelvic osteotomy is a technically demanding and complex surgery with many risks, including high blood loss, inability to obtain adequate fixation, and nonunion. In addition to the high surgical risk, the possibility of persistent GI symptoms after pelvic osteotomy also remains.^8,9^ No cases of a colostomy for the definitive treatment of refractory constipation or outlet obstruction in OI have been reported.^3,10^

The genotypic and phenotypic heterogeneity of OI make it challenging to study and treat. Although great progress has been made in understanding genetic and metabolic aspects of the disease over the past several few decades, notable opportunity remains for the potential role of pharmacologic interventions or gene therapy. The rarity of the disease paired with the high variability in clinical manifestations often leads to anecdotal treatment recommendations.

Gastrointestinal issues are common and also one of the leading causes of mortality in patients with OI.^11^ Pelvic deformities are also commonly seen in severe cases of OI, and these patients are typically poor candidates for complex pelvic reconstruction procedures. Our cases highlight colostomy as a possible treatment option for patients with OI who suffer from refractory constipation due to mechanical colonic outlet obstruction related to their pelvic deformity. Although colostomy may seem like an extreme treatment for chronic constipation, in patients with severe OI with chronic abdominal pain and difficulties eating, this procedure can have good clinical outcomes and high patient satisfaction, as seen in our two patients.
